# Application of Array Imaging Algorithms for Water Holdup Measurement in Gas–Water Two-Phase Flow Within Horizontal Wells

**DOI:** 10.3390/s25154557

**Published:** 2025-07-23

**Authors:** Haimin Guo, Ao Li, Yongtuo Sun, Liangliang Yu, Wenfeng Peng, Mingyu Ouyang, Dudu Wang, Yuqing Guo

**Affiliations:** 1 College of Geophysics and Petroleum Resources, Yangtze University, Wuhan 430100, China; ghm@yangtzeu.edu.cn (H.G.); 2022720524@yangtzeu.edu.cn (Y.S.); 2024710438@yangtzeu.edu.cn (L.Y.); 2024710433@yangtzeu.edu.cn (W.P.); 2024720502@yangtzeu.edu.cn (M.O.); 2023720526@yangtzeu.edu.cn (D.W.); 2023710415@yangtzeu.edu.cn (Y.G.); 2 Key Laboratory of Exploration Technology for Oil and Gas Resources of Ministry of Education, Yangtze University, Wuhan 430100, China

**Keywords:** capacitance array tool, horizontal well, gas–water two-phase flow, water holdup, imaging algorithm application

## Abstract

Gas–water two-phase flow in horizontal and inclined wells is significantly influenced by gravitational forces and spatial asymmetry around the wellbore, resulting in complex and variable flow patterns. Accurate measurement of water holdup is essential for analyzing phase distribution and understanding multiphase flow behavior. Water holdup imaging provides a valuable means for visualizing the spatial distribution and proportion of gas and water phases within the wellbore. In this study, air and tap water were used to simulate downhole gas and formation water, respectively. An array capacitance arraay tool (CAT) was employed to measure water holdup under varying total flow rates and water cuts in a horizontal well experimental setup. A total of 228 datasets were collected, and the measurements were processed in MATLAB (2020 version) using three interpolation algorithms: simple linear interpolation, inverse distance interpolation, and Lagrangian nonlinear interpolation. Water holdup across the wellbore cross-section was also calculated using arithmetic averaging and integration methods. The results obtained from the three imaging algorithms were compared with these reference values to evaluate accuracy and visualize imaging performance. The CAT demonstrated reliable measurement capabilities under low- to medium-flow conditions, accurately capturing fluid distribution. For stratified flow regimes, the linear interpolation algorithm provided the clearest depiction of the gas–water interface. Under low- to medium-flow rates with high water content, both inverse distance and Lagrangian methods produced more refined images of phase distribution. In dispersed flow conditions, the Lagrangian nonlinear interpolation algorithm delivered the highest accuracy, effectively capturing subtle variations within the complex flow field.

## 1. Introduction

Given the intricate nature of multiphase fluid flow in inclined and horizontal wells, combined with the constraints of conventional logging tools in both measurement and interpretation, many researchers have increasingly turned to advanced array logging instruments to address these challenges. Array logging instruments can precisely measure a broad range of fluid properties, including key parameters such as temperature, pressure, velocity, water holdup, gas holdup, and density. Accurate measurement of these parameters is essential for understanding the dynamics of downhole multiphase flow. In practice, array logging is commonly used to evaluate and measure critical parameters such as water holdup, apparent fluid velocity, phase velocity, and mixing velocity. Among these, the accurate measurement of water holdup is a core step in monitoring oil and gas well production. By tracking the variations in oil, gas, and water phases, researchers can effectively address the challenge of dynamically monitoring the ratios of oil, gas, and water production across different formations during production logging.

Water holdup measurement studies are of great significance in exploring multiphase fluid flow patterns and interpreting production logging profiles. The following methods are commonly used to measure water holdup: time domain reflectometry (TRD) [1,2,3], the capacitance method [4], the conductivity method [5,6,7], the tomography method [8,9], the ray method [10], the ultrasonic method [11,12,13,14], optical probe technology [15,16], and optical fiber sensing technology [17,18,19,20]. The multiple array production suite (MAPS) is the second generation of production logging instruments developed in recent years. When combined with imaging algorithms, it provides a more intuitive representation of the downhole distribution of oil, gas, and water. In research on water holdup imaging algorithms for oil–water two-phase flow, Shi et al. [21] conducted a detailed comparison of methods such as the average value method, layered interface method, imaging method, and integral method for calculating water holdup in low-level wells. The results showed that under low-water-content conditions, the imaging method offers the highest accuracy and captures the oil–water interface distribution the most precisely. However, when water content is high, the layered interface method performs better, with less error, more accurately reflecting the oil–water layering phenomenon under these conditions. Additionally, Li et al. [22] analyzed the applicability of the capacitance array tool (CAT) through oil–water two-phase physical simulation experiments and proposed two new methods for calculating wellbore holdup: the area averaging method and the micro-element integration method. The results indicate that the micro-element integration method meets the needs of field applications due to its consideration of the instrument’s cross-sectional area, achieving higher calculation accuracy with a relative error of less than 9%. Furthermore, Song [23] et al. conducted a study on water holdup imaging of oil–water two-phase fluids in horizontal wells using CAT data. Their research demonstrated that the ordinary kriging interpolation algorithm performs effectively in this application, accurately capturing the flow pattern of the oil–water two-phase fluid and the overall distribution of water holdup at the wellbore interface. Meanwhile, Niu [24] explored the applicability of Gaussian radial basis interpolation algorithms and linear interpolation algorithms under different total flow, well deviation, and water content conditions. The results indicated that the linear interpolation algorithm achieved the smallest error in calculating water holdup, showing a high degree of consistency with experimental data. Additionally, the linear interpolation algorithm accurately reflected the flow pattern distribution in the wellbore, highlighting its unique advantages in multiphase fluid imaging.

Advancements in monitoring technology have significantly improved the accuracy of multiphase flow holdup measurements. In this domain, MAPS have been widely adopted in oil and gas production processes both domestically and internationally. This system has become one of the key tools for production monitoring in oil and gas wells, owing to its precise data acquisition and analysis capabilities. Additionally, by integrating various advanced instruments such as the capacitance array tool (CAT), resistive array tool (RAT), and spinner array tool (SAT), researchers can measure the distribution of gas and water phases in detail. This combined logging technology not only excels in single-phase measurements but also provides comprehensive monitoring results under multiphase flow conditions.

In this study, the response characteristics of the CAT were analyzed in detail using an experimental setup. To ensure data accuracy and reliability, three interpolation algorithms were employed: the simple linear interpolation algorithm, the inverse distance interpolation algorithm, and the Lagrange interpolation algorithm. These algorithms offer multiple technical approaches for data processing and water holdup imaging, ensuring the accuracy and representativeness of the experimental results. The focus of the study is to analyze the two-dimensional characteristics of gas–water two-phase flow in horizontal wells under different total flow and varying water contents. Through in-depth analysis of the experimental data, the study reveals the gas–water distribution patterns under these complex flow conditions. Ultimately, the study successfully imaged the water holdup, providing a visual representation of the dynamic distribution of fluids in the wellbore, and offering a practical reference for oil and gas field production monitoring. The results indicate that, through experimental methods and the comprehensive application of multiple interpolation algorithms, this study effectively analyzes the two-dimensional characteristics of gas–water two-phase flow in horizontal wells under various conditions and achieves accurate water holdup imaging. This research provides strong support for improving the accuracy of multiphase flow monitoring, which is of great significance for the future monitoring and management of oil and gas field production.

## 2. Methodology

### 2.1. Experimental Summary

**Figure 1:**
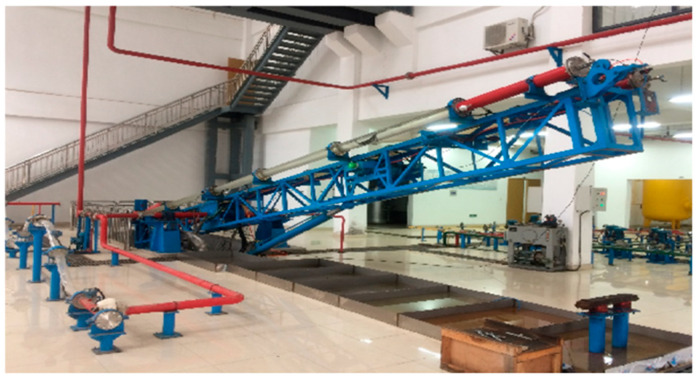
A photo of the Oil and Gas multiphase flow simulation laboratory.

As shown in Figure 1, this experiment was conducted in the Oil and Gas Multiphase Flow Simulation Laboratory for horizontal wells with large gradients at Yangtze University in Hubei Province.

**Figure 2:**
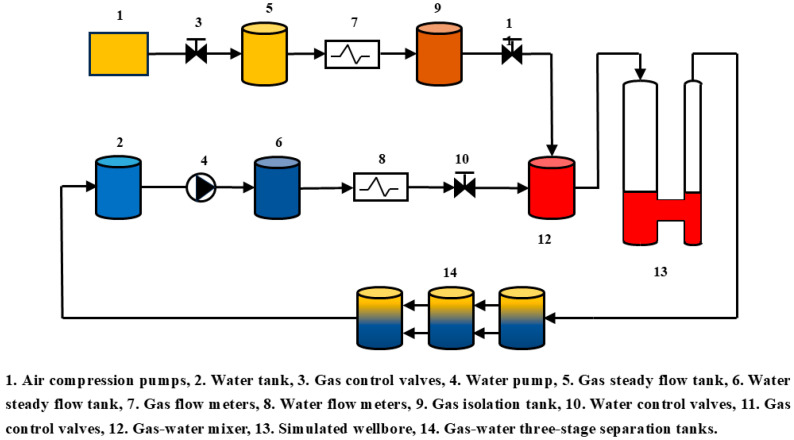
Schematic of multiphase flow experimental set.

The total length of the simulated wellbore is 12.0 m, including a 10.0 m long transparent glass tube with two outer diameter sizes: 139.7 mm and 177.8 mm. To simulate multiphase flow in wellbores of different sizes, the transparent glass wellbore with an outer diameter of 159 mm was selected. This design allows the study to more accurately replicate the flow environment of actual wellbores and better understand the effect of size variation on multiphase flow behavior by comparing flow characteristics in different wellbore sizes. The use of transparent glass significantly enhances the observability of the experiments, enabling researchers to directly observe the fluid flow patterns in the simulated wellbore. This feature greatly facilitates experimental operations and data collection, providing an important visual reference for analyzing the dynamic characteristics of multiphase flow. Additionally, the hydraulic system that simulates the wellbore is one of the core components of this setup. The system consists of a support arm and a pressure pump, with its primary function being to secure the wellbore position and adjust the well deviation. Using the hydraulic system’s telescopic support arm, researchers can simulate fluid flow under different well inclination conditions, enabling a more comprehensive analysis of multiphase fluid behavior and distribution under varying deviation, as shown in Figure 2.

Due to the need for the experiment to be carried out in a safe environment, this experiment was conducted at normal temperature and pressure (20 °C, 0.101 MPa), using air and tap water as the flow media to simulate downhole gas and formation water, respectively. Specifically, the density of the air was 1.205 $kg/m^{3}$, with a viscosity of 1.81 × 10^−2^ mPa·s, while the density of the tap water was 998 $kg/m^{3}$, with a viscosity of 1.16 mPa·s. The experiment encompassed a variety of operating conditions, including different well deviation, total flow, and water contents, as shown in Table 1. These variables were established to comprehensively analyze the behavior and characteristics of gas–water two-phase flow under various complex conditions. In total, 228 datasets were collected during the experiment (including replicated experimental data).

### 2.2. CAT Instrument Description and Measurement Principle

**Figure 3:**
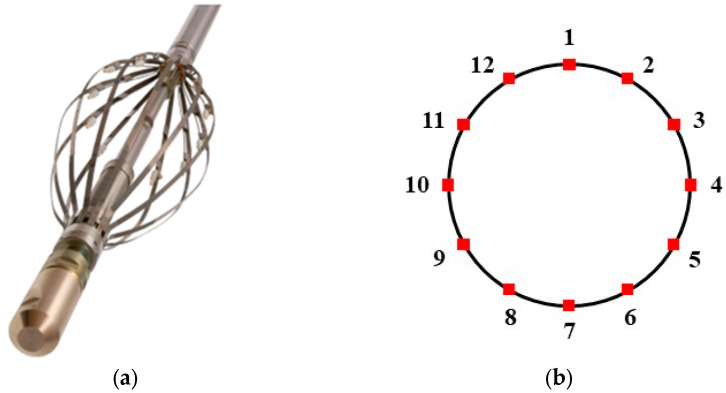
Schematic diagram of the capacitive array tool (**a**) and sensor array cross-section (**b**).

The capacitance array tool (CAT) consists of a main body and 12 freely retractable arms. As shown in Figure 3a, each arm is spring-loaded and the tool extends into an umbrella-like shape, fitting snugly against the wellbore wall to ensure adaptability to a wide range of wellbore sizes and shapes. Each arm is equipped with a miniature capacitive sensor, positioned equidistantly from the inner surface of the wellbore. In this configuration, the sensors form a monitoring circle within the wellbore, as shown in Figure 3b, providing full coverage of the wellbore’s inner surface. This design enables the CAT to accurately monitor the water holdup in real time, ensuring the precision and reliability of the data, regardless of changes in wellbore shape or size. The CAT features spring-loaded arms that fully expand during operation and conform closely to the inner wall of the transparent glass tube, minimizing interference with the central flow region. Moreover, given the small physical size of the CAT and the limited cross-sectional area it occupies, most of the gas–water two-phase flow was able to pass through the gaps between the sensor arms, resulting in minimal disruption to the overall flow field.

**Figure 4:**
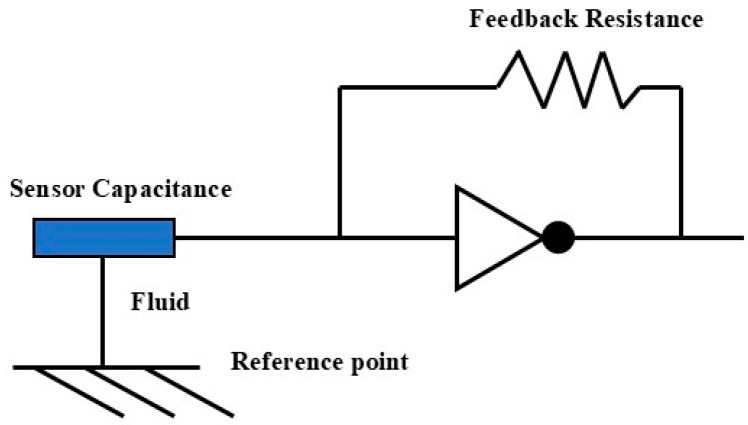
Schematic diagram of sensor simplified circuit.

The operating principle of the CAT is based on the differences in dielectric constants between various fluid phases. The oil, gas, and water phases exhibit significant variations in their dielectric constants, providing a theoretical foundation for the accurate identification of fluid phase states. In the experiments, the relative dielectric constants of the gas and water phases are 1 and 80, respectively, meaning they exhibit markedly different capacitance properties in an electric field. The relative permittivity of the oil phase falls between that of the gas and water phases, so the capacitance values measured by the probe in the oil phase are typically closer to those of the gas phase. Each miniature capacitance sensor converts the dielectric constant of the surrounding fluid into a frequency signal via a connected measurement circuit, as shown in Figure 4. This frequency signal directly reflects the dielectric properties of the fluid near the sensor, aiding in the identification of the phase. Specifically, when the probe is in the gas phase, the measured capacitance value is low, whereas when it is in the water phase, the capacitance value is high. By analyzing these signals, the instrument can accurately determine the fluid phase distribution at various locations within the wellbore. This principle of operation, based on differences in dielectric constants, enables the array capacitance holdup meter to effectively identify the distribution of oil, gas, and water phases within the wellbore.

**Figure 5:**
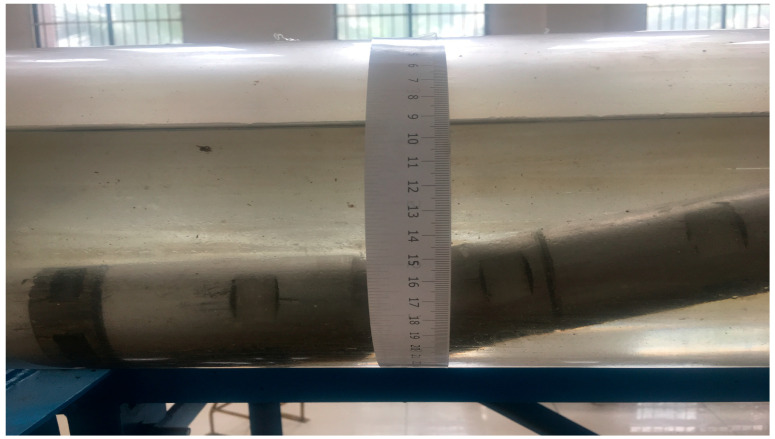
A physical photo of the gas–water two-phase holdup scale.

Before the experiment began, a prepared graduated scale was attached to the simulated wellbore to ensure measurement accuracy. To minimize potential errors from the scale calculation, the zero point of the scale was carefully aligned with the highest point of the simulated wellbore, as shown in Figure 5. This precise alignment of the scale helped to obtain more accurate measurement data during the experiment.

**Figure 6:**
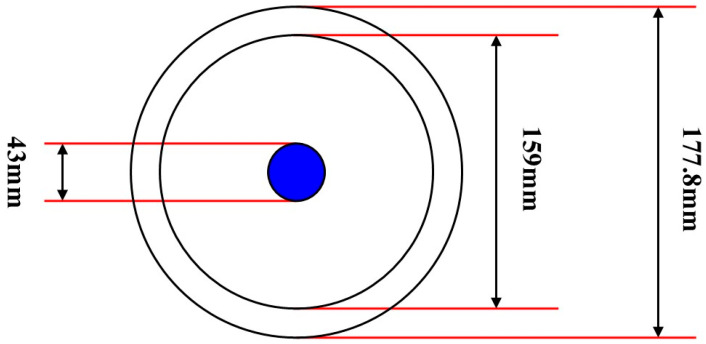
Schematic diagram of the simulated wellbore structure.

In this experiment, the outer diameter of the multiphase flow simulation wellbore was measured to be 177.8 mm, while the inner diameter was 159.0 mm. Additionally, according to the instrumentation specifications, the outer diameter of the array capacitance holdup meter used was 43 mm, as shown in Figure 6.

**Figure 7:**
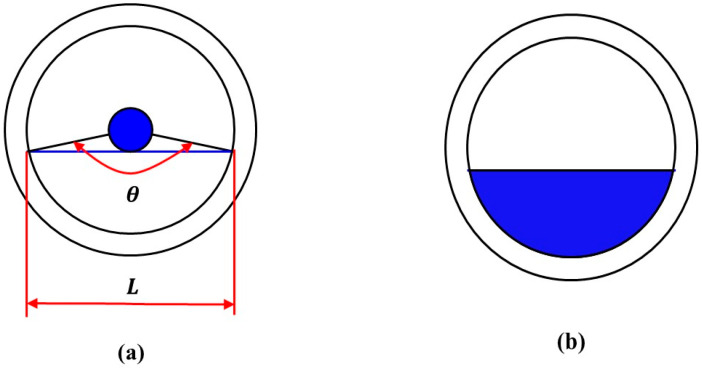
Schematic diagram of calculating gas–water two-phase holdup. (**a**) Schematic diagram of the graduated scale calculating the water holdup. (**b**) the water phase occupies the area of the wellbore cross-section.

The corresponding arc length (L) of the water phase in the simulated wellbore was measured using scale photographs. These arc length data served as the foundation for subsequent quantitative analysis. The corresponding circumferential angle (θ) was then calculated using Equation (1), a crucial step for accurately determining the distribution of the water phase within the wellbore. By analyzing the intersection of the circumferential angle (θ) with the wellbore wall, the interface height of the water phase was successfully identified. Once the interface height of the water phase was determined, the cross-sectional area occupied by the water phase in the wellbore was calculated using Equation (2), as illustrated in Figure 7. This calculation is critical, as the cross-sectional area of the water phase directly influences the final measurement of water holdup. Finally, by comparing the cross-sectional area of the wat phase with the effective cross-sectional area of the wellbore, the water holdup was calculated using Equation (3).

(1) $$\theta =\frac{L}{C}\times 360$$

(2) $$S_{w}=\frac{\theta }{360{}^{\circ}}\times S-\frac{1}{2}r^{2}\sin\theta$$

(3) $$Y_{w}=\frac{S_{w}}{S}$$

where $Y_{w}$ is the water holdup; S is the cross-sectional area of simulated wellbore, $m^{2}$; $S_{w}$ is the cross-sectional area of simulated wellbore occupied by the water phase, $m^{2}$; θ is the central angle corresponding to the area of the water phase, °; L is the length of the arc corresponding to θ, mm; and C is the circumference of simulated wellbore, mm.

### 2.3. Analysis of CAT Response Characteristics

**Figure 8:**
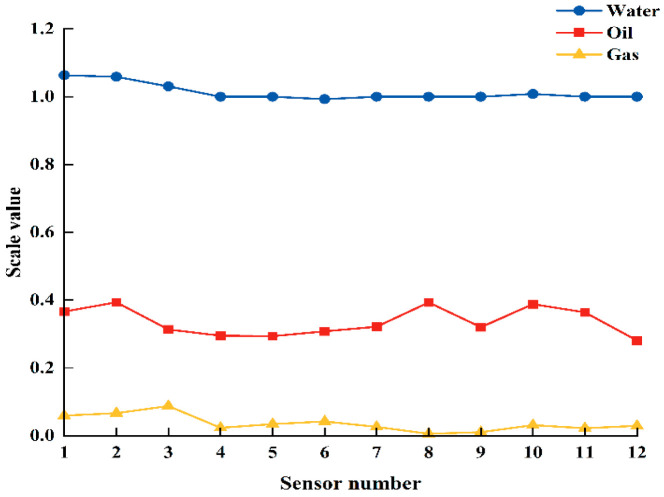
Schematic of CAT single-phase fluid scale value.

Due to the varyious positions of the instrument’s 12 sensors, different readings are generated during the measurement process, reflecting the complex distribution of multiphase fluids within the wellbore. After measuring the water content, these readings must be further processed to obtain an accurate value for the water holdup. The first step involves converting the normalized value (NCAP) from the CAT measurements into the instrument’s response value (RAW), which is critical in calculating the water holdup. This conversion is based on specific formulas: when the NCAP value is greater than or equal to 0.2, the response value RAW is calculated using Equation (4), and when the NCAP value is less than 0.2, the response value RAW is calculated using Equation (5). Then, 12 localized holdup values were calculated based on the CAT readings in the three-phase fluids (oil, gas, and water), as shown in Figure 8. These values serve as key reference data for ensuring accurate measurements throughout the experiment.

(4) $$RAW=Gas-\frac{NCAP\times (Gas-Oil)}{0.2}$$

(5) $$RAW=Oil-\frac{\left(NCAP-0.2\right)\times (Oil-Water)}{0.8}$$

The water holdup values from the all CAT sensors were normalized to the Equation (6).

(6) $$Y_{iw}=\frac{m_{i}-m_{ig}}{m_{iw}-m_{ig}}$$

where $Y_{w}$ is the water holdup measured by the *i*th sensor, i = 1, 2, ⋯, 12; $m_{i}$ is the measured value of the *i*th sensor; $m_{ig}$ is the scale value of the *i*th sensor in gas; and $m_{iw}$ is the scale value of the *i*th sensor in water.

## 3. Water Holdup Calculation Methods

### 3.1. Experimental Data Analysis

**Figure 9:**
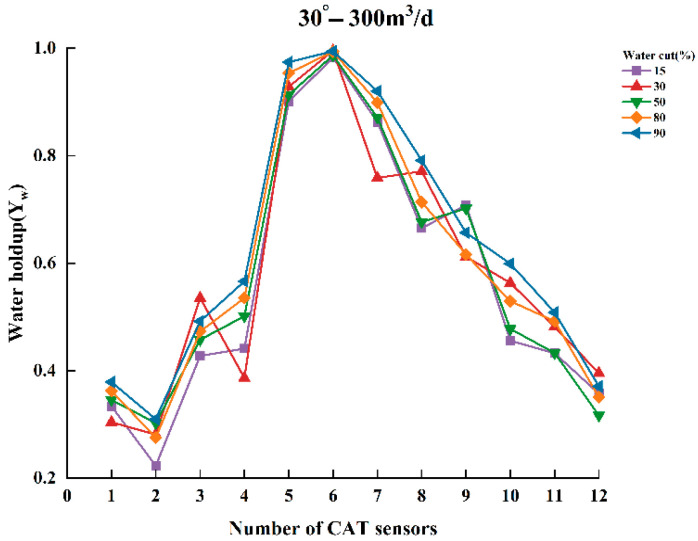
Response of water holdup from 12 sensors when the well deviation is 30° and the total flow is 300 m^3^⁄d.

**Figure 10:**
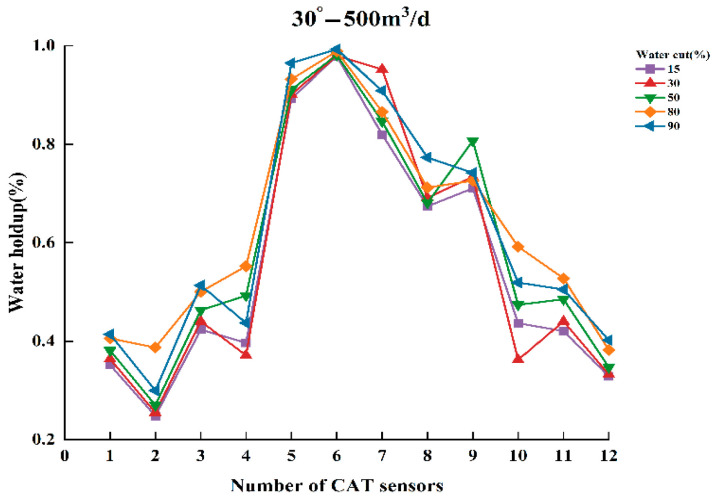
Response of water holdup from 12 sensors when the well deviation is 30° and the total flow is 500 $m^{3}/d$.

**Figure 11:**
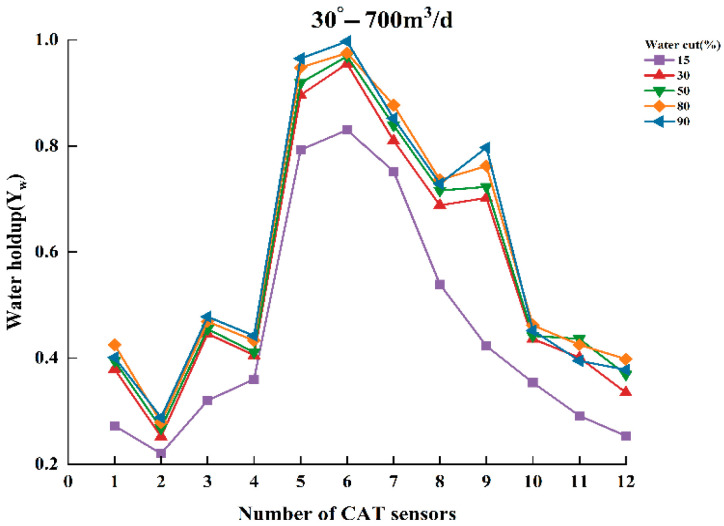
Response of water holdup from 12 sensors when the well deviation is 30° and the total flow is 700 $m^{3}/d$.

**Figure 12:**
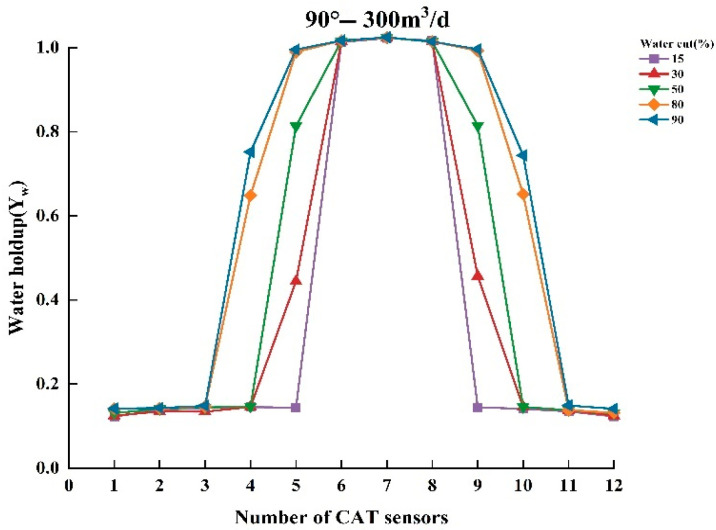
Response of water holdup from 12 sensors when the well deviation is 90° (horizontal) and the total flow is 300 $m^{3}/d$.

**Figure 13:**
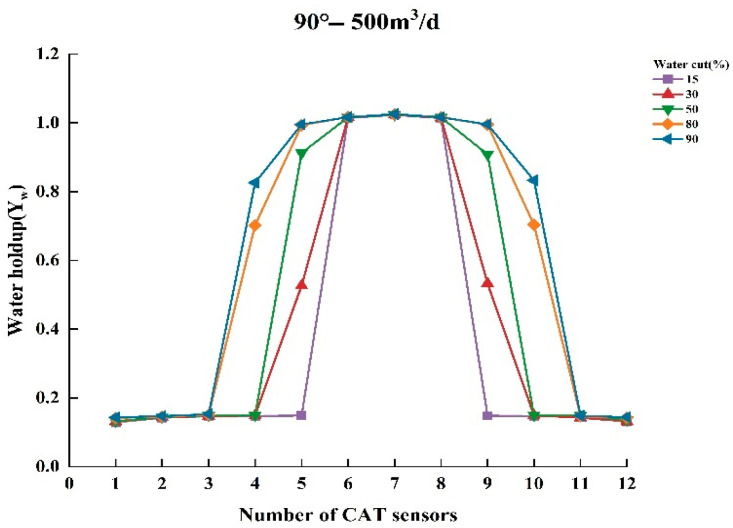
Response of water holdup from 12 sensors when the well deviation is 90° (horizontal) and the total flow is 500 $m^{3}/d$.

**Figure 14:**
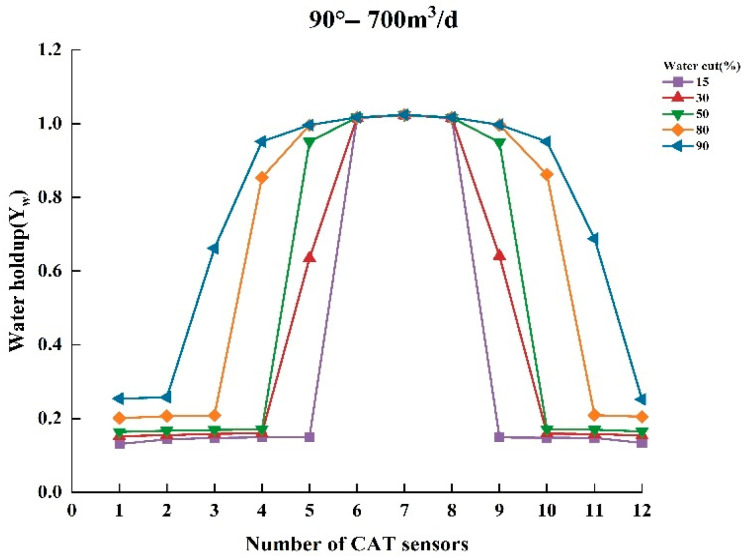
Response of water holdup from 12 sensors when the well deviation is 90° (horizontal) and the total flow is 700 $m^{3}/d$.

To thoroughly analyze the variation in water holdup in multiphase flow, experiments were conducted to measure the normalized water holdup values of gas and water phases using CAT under different experimental conditions. The experiments covered several combinations of key parameters, including total flow of 300, 500, and 700 m^3^/d; water contents of 15%, 30%, 50%, 80%, and 90%; and well deviation of 30° and 90° (relative to the vertical direction). Under these conditions, experimental data were collected and analyzed to reveal the dynamic characteristics of water holdup under varying experimental conditions. Figure 9, Figure 10, Figure 11, Figure 12, Figure 13 and Figure 14 illustrate the relationship between water holdup, water content, well deviation, and total flow in the experimental data.

When the well deviation was set to 30°, the gas and water phases were significantly influenced by their density differences at total flow of 300 m^3^/d and 500 m^3^/d. The less dense gas phase tended to occupy the upper region of the fluid, while the denser water phase concentrated in the lower region. As the water content gradually increased, more sensors were positioned in the aqueous phase, resulting in a corresponding increase in the measured water holdup. As the cross-sectional area of the wellbore occupied by the water phase expanded, the area occupied by the gas phase decreased accordingly.

When the well deviation is set to 90°, the wellbore is in a horizontal state. At total flows of 300 m^3^/d and 500 m^3^/d, the water holdup measurements from the CAT instrument become more pronounced, as shown in Figure 12 and Figure 13. The fluid distribution exhibits a typical stratified flow pattern, with the gas phase concentrated at the top of the wellbore due to its lower density, while the water phase settles at the bottom. This stratified flow pattern provides a clear representation of the CAT instrument’s measurements, allowing the data from individual sensors to display distinct response characteristics along the longitudinal axis. At low water contents (15%, 30%), most CAT sensors are positioned in the gas phase, and thus primarily capture the flow characteristics of the gas. However, as the water content increases, the proportion of the water phase in the wellbore expands, and the number of CAT sensors in the water phase rises. This indicates that the coverage of the water phase is progressively expanding, allowing the CAT sensors to capture more extensive flow information from the water phase.

The fluid distribution in inclined (30°) and horizontal (90°) wells is analyzed in detail when the total flow reached 700 m^3^/d under high-flow conditions. The results show that in both wellbore inclination scenarios, most of the CAT sensors are located in the aqueous phase, with only a few sensors detecting the presence of the gas phase. This indicates that the gas phase distribution in the wellbore is significantly reduced under high-flow conditions, with the water phase occupying the majority of the wellbore cross-section. The discrepancy between the CAT sensor readings and the expected actual response in this flow condition arises from the fact that most of the sensors are positioned in the water phase. As a result, the CAT may overestimate the water holdup, failing to accurately reflect the actual distribution of the gas phase. This discrepancy suggests that under high-flow conditions, CAT measurements may be influenced by the fluid distribution characteristics, leading to reduced measurement accuracy.

### 3.2. Water Holdup Calculation Methods

In this experiment, due to the inability to directly obtain the water holdup of the wellbore cross-section, three different methods were used to calculate the overall water holdup of the wellbore: arithmetic average algorithm, integration algorithm, and imaging algorithm. Each of these methods has its own characteristics and can provide reliable water holdup calculations in different scenarios.

The arithmetic averaging algorithm is a straightforward method that calculates the overall water holdup by weighting and averaging the data from the CAT’s 12 sensors. This method is simple and intuitive, making it suitable for quick estimation of wellbore water holdup under the stratified flow conditions.

**Figure 15:**
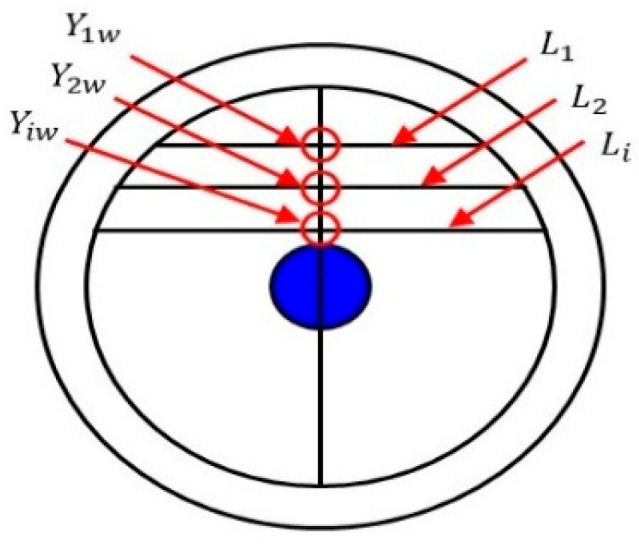
Schematic diagram of integration method.

The integration algorithm is more detailed and precise. This method calculates the water holdup $Y_{w}$ by projecting the measurements from the 12 sensors onto the longitudinal axis of the wellbore and linearly interpolating the water holdup between neighboring sensors. In this step, the cross-sectional area of the wellbore is assumed to consist of n parallel line segments, with each value of water holdup corresponding to a segment of length L. The method is based on the assumption that the water holdup of a wellbore with a value greater than a certain threshold is the same as that of a line segment. By integrating $Y_{iw}$ and $L_{i}$ for water holdup values greater than a given threshold (assumed to be 0.51), the ratio of the integration result to the wellbore cross-sectional area S is used as the final calculated water holdup value, as shown in Figure 15. This method not only accounts for data variations between sensors but also provides an accurate estimation of water holdup through the integration process. The calculation formula is:

(7) $$Y_{w}=\int_{i=1}^{n}\frac{Y_{iw}\times L_{i}}{S}$$

where $Y_{iw}$ is the water holdup of the *i*th CAT sensor, $L_{i}$ is the water holdup corresponding to a length L segment of the *i*th CAT sensor, and i = 1, 2, ⋯, 12.

The imaging algorithm, in contrast, utilizes imaging of the fluid distribution in the wellbore, combined with data from the CAT sensors, to directly visualize the spatial distribution of water holdup using an interpolation algorithm. This approach is especially suited for situations where detailed knowledge of the fluid phase distribution is required, providing intuitive data support for water holdup measurements.

## 4. Imaging Algorithms of Gas–Water Two-Phase Flow

### 4.1. Data Preprocessing of CAT Measurement

**Figure 16:**
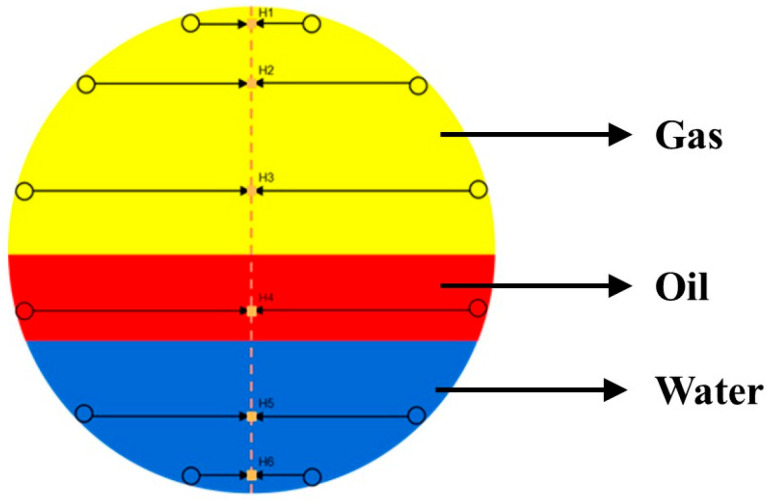
Schematic diagram of 12-sensor wellbore distribution (symmetrical).

During the experiment, it is crucial to ensure the accuracy and consistency of the sensor data, particularly due to the potential cornering of the CAT within the simulated wellbore. To properly process and analyze the CAT sensor data, it is necessary to transform the sensor positions into a right-angle coordinate system while fully accounting for the CAT’s cornering. When the turning angle is 0° or a multiple of 15°, the 12 sensors of the CAT will exhibit a symmetrical distribution along the longitudinal center axis of the wellbore, as shown in Figure 16 (yellow represents the gas phase, red represents the oil phase, and blue represents water). This symmetrical distribution means that the projection points of the symmetrically distributed sensors on the center axis will coincide at the same point. Therefore, to ensure data accuracy, Equation (8) must be used to calculate the coordinates of these projection points.

(8) $$X_{H_{i}}=\frac{r\times \cos\left(\frac{2i-1}{12}\pi \right)}{2}$$

where $X_{H_{i}}$ is the transverse coordinate of the projection point of the CAT sensor on the center axis; r is the outer diameter of the CAT, mm; and i = 1, 2, ⋯, 12.

Considering sensors with the same projection point as a single point, the water holdup value at the projection point is calculated using the following formula:

(9) $$Y_{H_{i}\;}=\frac{Y_{wi}+Y_{w(13-i)}}{2}$$

where $Y_{H_{i}}$ is the approximate water holdup value at the projection point $H_{i}$, and $Y_{wi}$ is the water holdup response value measured by the $i^{th}$ sensor, where i = 1, 2, ⋯, 6.

**Figure 17:**
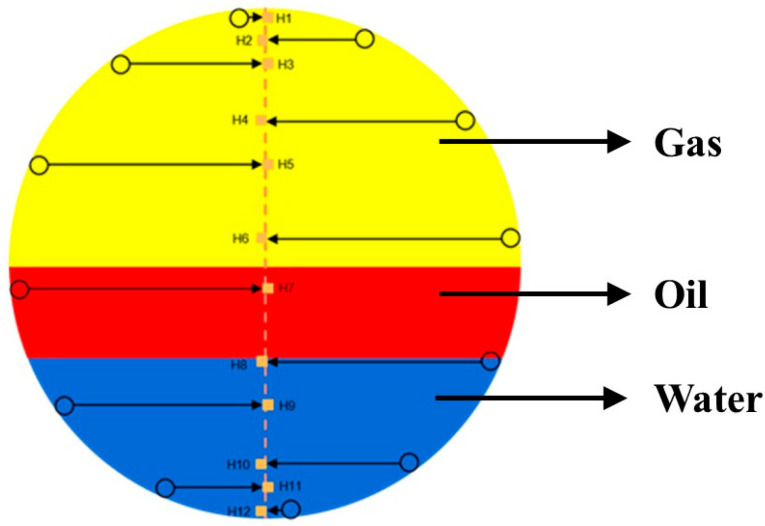
Schematic diagram of sensors wellbore distribution (asymmetrical).

When the CAT is rotated by an angle θ that is not a multiple of 15° in the simulated wellbore, the 12 sensors will be asymmetrically distributed, as shown in Figure 17 (yellow represents gas phase, red represents oil phase, and blue represents water). In this case, the position of each sensor shifts relative to the longitudinal center axis of the wellbore and the positions of the other sensors. When the sensors are asymmetrically distributed, the calculation method used for symmetric distribution may not accurately reflect the actual water holdup and fluid distribution. Therefore, the calculation method must be adjusted to account for this asymmetry when performing coordinate transformation of sensor positions and data analysis.

First, the angle of each sensor in the simulated wellbore is calculated using Equation (10). By calculating the angle for each sensor, its position within the wellbore cross-section can be accurately determined, providing the foundation for the subsequent projection calculations. Next, the projection coordinates of each sensor on the central axis of the wellbore cross-section are calculated using Equation (11). This step projects the sensor positions onto the central axis, offering a more intuitive representation of the sensor data distribution within the wellbore. Both the determination of sensor angles and projection coordinates provide reliable data support for the final water holdup calculation.

(10) $$\alpha =\frac{2i-1}{12}\pi +\theta$$

(11) $$X_{H_{i}}=r\times \frac{\cos\alpha }{2}$$

where α is the turning angle of the CAT in the simulated wellbore, °, $X_{H_{i}}$ is the transverse coordinate of the projection point of the *i*th sensor on the mid-axis of the wellbore cross-section, and r is the outer diameter of the CAT, with i = 1, 2, ⋯, 12.

### 4.2. Simple Linear Interpolation (SLI)

**Figure 18:**
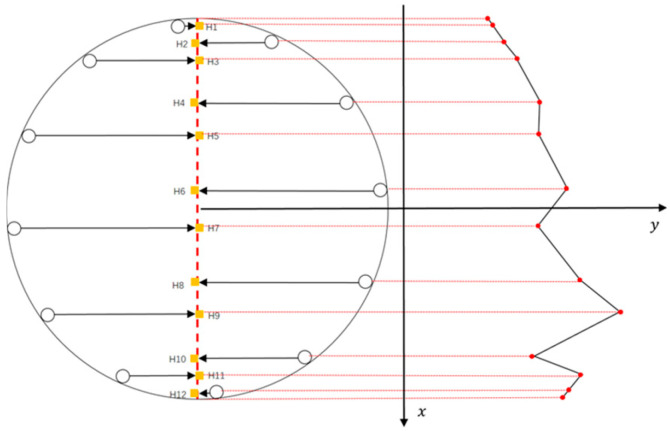
Schematic diagram of the simple linear interpolation.

In the simulated wellbore, the fluid flow pattern is primarily influenced by gravity, causing the heavier phase (water) to settle at the bottom of the wellbore, while the lighter phase (gas) is distributed above the water, forming a distinct interface between the two. The principle of the simple linear interpolation algorithm is to project the response values of the 12 CAT sensors linearly onto the central axis of the wellbore based on the sensors’ distance from the axis, resulting in 12 non-equally spaced projection points. The response values of each sensor in the cross-section are converted through this linear projection into holdup values at corresponding locations on the central axis. These holdup values at the projection points approximate the fluid holdup distribution in the simulated wellbore, as shown in Figure 18. The specific holdup calculation formulas, shown in Equations (12) and (13), are based on using the projection points of the CAT sensors as the horizontal coordinates and the holdup measurements at those points as the vertical coordinates.

(12) $$\frac{Y_{w}\left(x\right)-Y_{H_{i}}}{Y_{H_{i+1}}-Y_{H_{i}}}=\frac{x-H_{i}}{H_{i+1}-H_{i}}$$

(13) $$Y_{w}\left(h\right)=\frac{Y_{H_{i+1}}-Y_{H_{i}}}{H_{i+1}-H_{i}}h-\frac{Y_{H_{i}}H_{i+1}-Y_{H_{i+1}}H_{i}}{H_{i+1}-H_{i}}$$

where $H_{i}$ is the projection point of the sensor, $Y_{H_{i}}$ is the response value at the sensor’s projection point, i = 1, 2, ⋯, 12, and $Y_{w}\left(h\right)$ are the holdup values at the projection position h, respectively.

### 4.3. Inverse Distance Weighted (IDW) Interpolation

The inverse distance weighted (IDW) interpolation algorithm is a widely used method for interpolating spatial data. The core principle of the algorithm is based on n known points in space, where each known point has a corresponding attribute value $Z_{i\;}(0\;<\;i\;\leq \;n)$. The algorithm provides an efficient way to estimate the attribute values of a discrete point $\left(x_{0},\;y_{0}\right)$, which exists in space but has unknown attribute values. The algorithm assumes that the attribute value Z of the unknown point is related to the attribute values of the known points by distance: the closer the distance, the closer the attribute values of the two points. The $k^{th}$ power of the inverse of the Euclidean distance between the known and unknown points is used as the weighting factor. With this weighting factor, a closer known point has a greater influence on the estimation of the attribute value of the unknown point. The parameter k is in the range 0 ≤ k < 3, with k = 2 being the most common choice.

IDW first calculates the Euclidean distance between the unknown point and all known points using Equation (14), then assigns a corresponding weight to each known point based on the inverse of the distance. The attribute values of all known points are then multiplied by their respective weight factors, and these weighted values are summed to obtain the estimated attribute value Z of the unknown point, as shown in Equation (15).

(14) $$D_{i}=\sqrt{{\left(x_{0}-x_{i}\right)}^{2}+{\left(y_{0}-y_{i}\right)}^{2}}$$

(15) $$Z=\frac{\sum_{i=1}^{n}{\left(D_{i}\right)}^{k}Z_{i}}{\sum_{i=1}^{n}\frac{1}{{\left(D_{i}\right)}^{k}}}$$

where Z is the attribute value to be determined at a point in space, $D_{i}$ is the distance between the unknown point and the *i*th known point in space, and $x_{i},\;y_{i}$ are the coordinate values of the points with known attribute values. The term $\frac{1}{D_{i}^{k}}$ is the computational weighting coefficient between the unknown point and the *i*th known point, where n is the number of sensors in the CAT (n = 12), and i = 1, 2, ⋯, n.

Based on the above theory, the value of the holdup at any point $\left(x_{i},\;y_{i}\right)$ in the simulated wellbore can be calculated as:

(16) $$Y_{w}=\frac{\sum_{i=1}^{n}\frac{Y_{wi}}{D_{i}^{k}}}{\sum_{i=1}^{n}\frac{1}{{\left(D_{i}\right)}^{k}}}$$

where $Y_{w}$ is the calculated holdup value at the coordinate point $\left(x_{i},\;y_{i}\right)$, and $Y_{wi}$ is the measured holdup value of the *i*th CAT sensor, with n = 12 and i = 1, 2, ⋯, n.

### 4.4. Lagrangian Nonlinear Interpolation (LNI)

The Lagrange nonlinear interpolation algorithm is a classical numerical analysis method widely used to approximate the function value of an unknown point given discrete data points. The core idea of the algorithm is to construct an overall polynomial relationship based on the known discrete numerical points, enabling interpolation for any unknown point in the plane. The advantage of this method is that it is not only suitable for the interpolation of linear data, but also effectively handles complex nonlinear data. Since Lagrange interpolating polynomials consider the overall relationship of all known points, they can more accurately capture both the overall trend and local variations in the data during interpolation. This makes the Lagrange nonlinear interpolation algorithm particularly effective when dealing with discrete data that exhibit complex relationships.

Assuming there are $\left(x_{1},\;y_{1}\right),\;\left(x_{2},\;y_{2}\right),\;\cdots ,\;(x_{n},\;y_{n})$ points in the coordinate system, and a function L(x) passes through these n points, the calculation formula is:

(17) $$L\left(x\right)=\sum_{j=1}^{n}y_{i}l_{j}(x)$$

(18) $$l_{j}\left(x\right)=\prod_{i=1}^{j}\frac{x-x_{i}}{x_{j}-x_{i}}$$

where L(x) is the Lagrange function, $x_{i}$ and $y_{i}$ are the horizontal and vertical coordinates of the *i*th point, and $l_{j}(x)$ is the polynomial fit calculated from the horizontal coordinates of the points with indices less than j.

**Figure 19:**
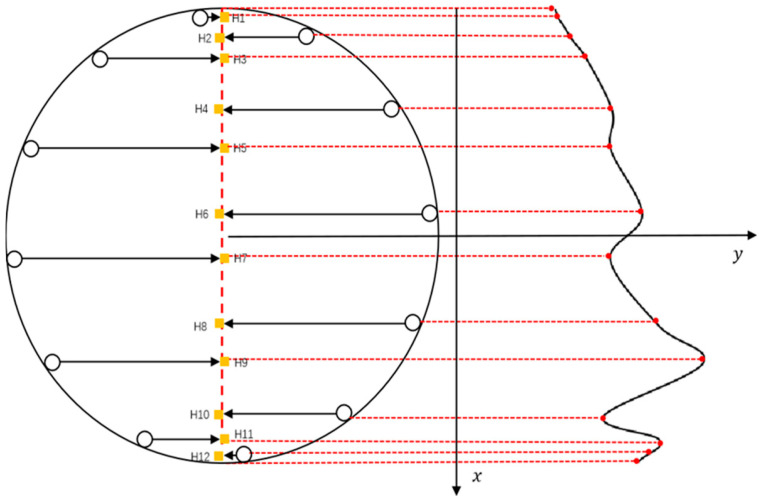
Schematic diagram of the Lagrange nonlinear interpolation.

According to the above theory, after substituting the coordinates of the 12 CAT sensors in the Cartesian coordinate system into Equations (18) and (19), the Lagrange function passing through all the points is calculated, as shown in Figure 19.

## 5. Results and Discussion

Table 2, Table 3 and Table 4 shows the water-holdup response values for each CAT sensor at a well deviation of 90°, with different total flow and varying water contents.

Using a total flow of 300 m^3^/d as an example, the water holdup in the wellbore was calculated using the arithmetic average method, the calculus integration method, and three imaging algorithms. The results are listed in Table 5 and Table 6. By comparing the results of these methods, it was found that the micrometric integration method provided the most consistent calculated water holdup value with the actual experimental data, making it the benchmark algorithm. The relative errors of the arithmetic mean method and the three interpolation algorithms were also calculated, and the imaging algorithms suitable for on-site production wells were identified.

**Figure 20:**
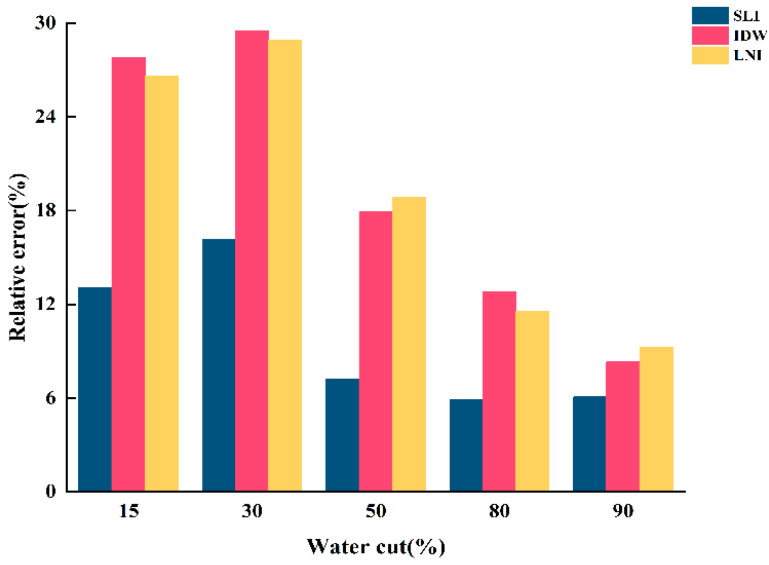
Relative error of water holdup calculated by the three algorithms for the total flow of 300 $m^{3}/d$.

**Figure 21:**
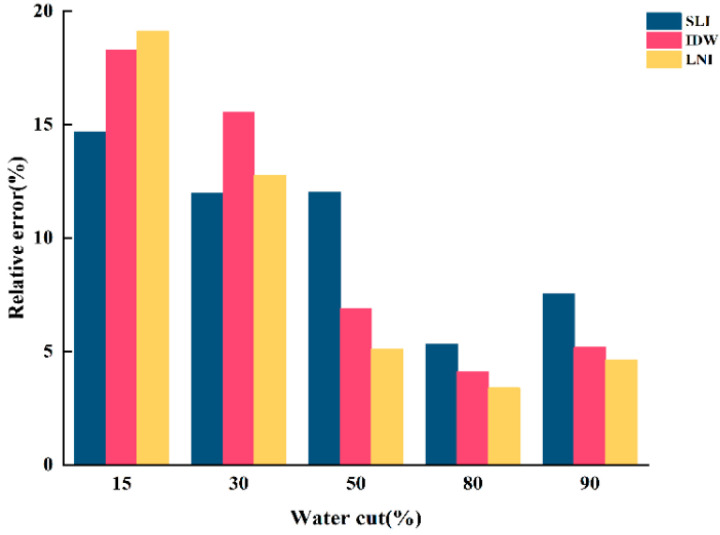
Relative error of water holdup calculated by the three algorithms for the total flow of 500 $m^{3}/d$.

**Figure 22:**
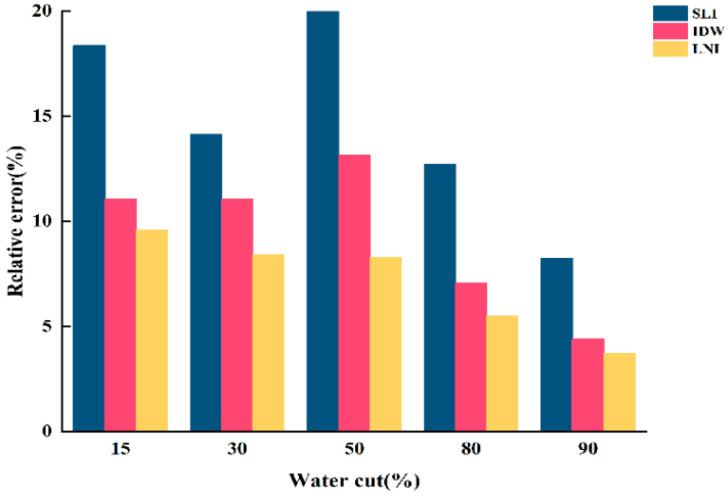
Relative error of water holdup calculated by the three algorithms for the total flow of 700 $m^{3}/d$.

To validate the accuracy of the water holdup values calculated by SLI, IDW, and LNI are consistent with actual experimental conditions. The relative errors between the water holdup values of the three algorithms and those calculated by the micrometric integration method were determined. The results are shown in Figure 20, Figure 21 and Figure 22.

When the total flow is 300 m^3^/d, the linear interpolation algorithm shows the lowest relative error, providing a more accurate reflection of the actual water holdup. In contrast, the inverse distance interpolation and Lagrangian interpolation algorithms exhibit relatively higher relative errors and poorer accuracy, failing to capture the actual water holdup distribution accurately. When the total flow is 500 m^3^/d, the simple linear interpolation algorithm continues to perform well under low-water-content conditions, delivering more accurate water holdup imaging results and maintaining a low relative error. However, as water content increases, the performance of the inverse distance interpolation and Lagrangian interpolation algorithms improves, with accuracy significantly increasing. In high-water-content conditions, both the inverse distance interpolation and Lagrangian interpolation algorithms more accurately capture the actual water holdup distribution, and their relative errors are greatly reduced. When the total flow is 700 m^3^/d, the relative error of the simple linear interpolation algorithm increases significantly, resulting in poor accuracy. Although the error decreases with increasing water content, its overall performance remains inferior to the other two algorithms, indicating poor applicability under high-flow conditions. At this point, the Lagrangian interpolation algorithm performs best, exhibiting the lowest relative error. This algorithm accurately captures changes in water holdup under high total flow and complex fluid distributions, making it the best choice for water holdup imaging under high-flow conditions. The inverse distance interpolation algorithm, while not as accurate as the Lagrangian interpolation algorithm, still achieves a high degree of accuracy and adequately reflects the actual water holdup distribution. Its relative error is second only to the Lagrangian interpolation algorithm, demonstrating strong adaptability under high-flow and high-water-content conditions.

**Figure 23:**
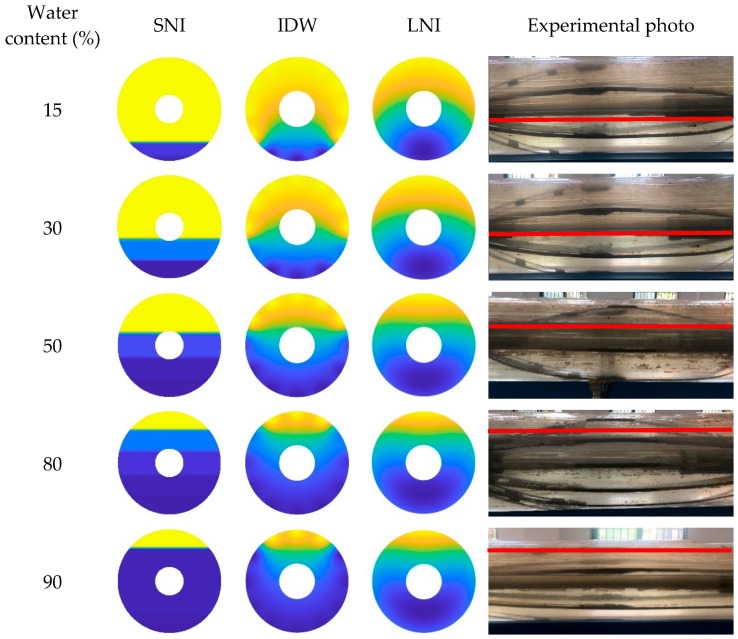
Water holdup imaging results and experimental photographs across varying water contents at a total flow of 300 m^3^/d.

**Figure 24:**
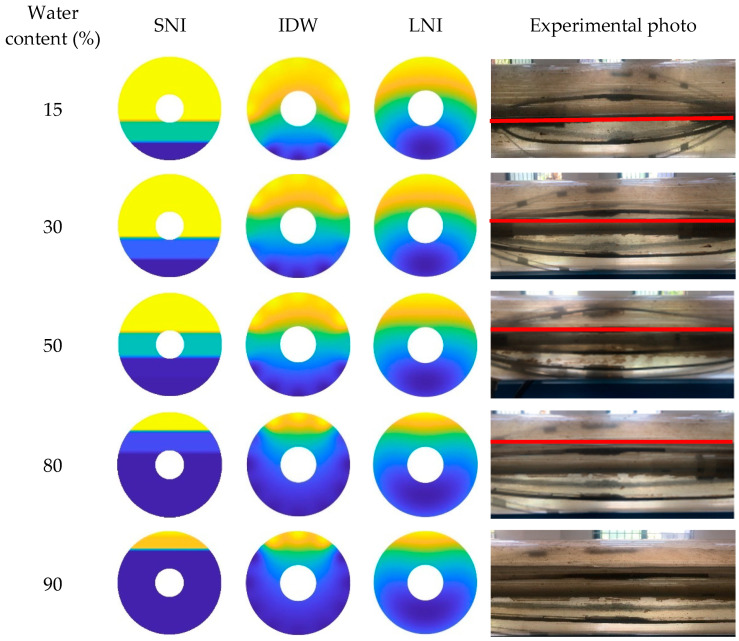
Water holdup imaging results and experimental photographs across varying water contents at a total flow of 500 m^3^/d.

**Figure 25:**
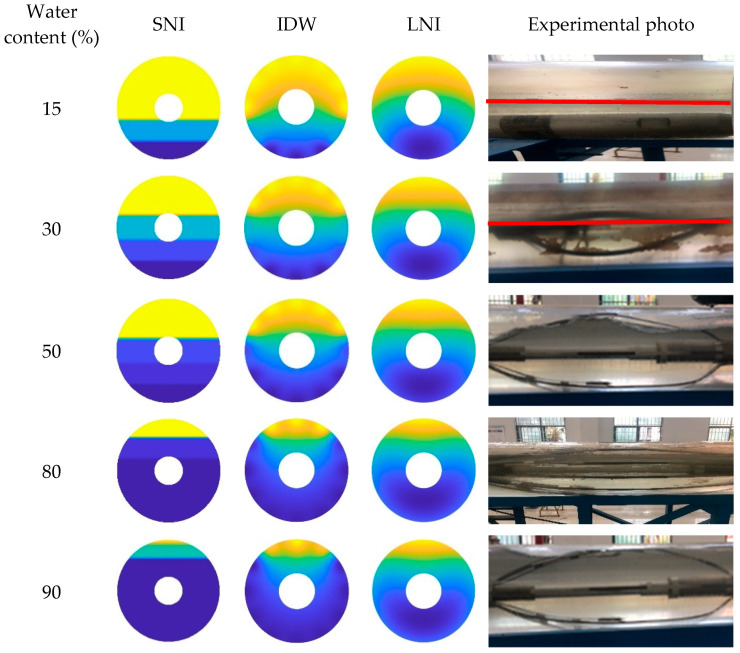
Water holdup imaging results and experimental photographs across varying water contents at a total flow of 700 m^3^/d.

**Figure 26:**
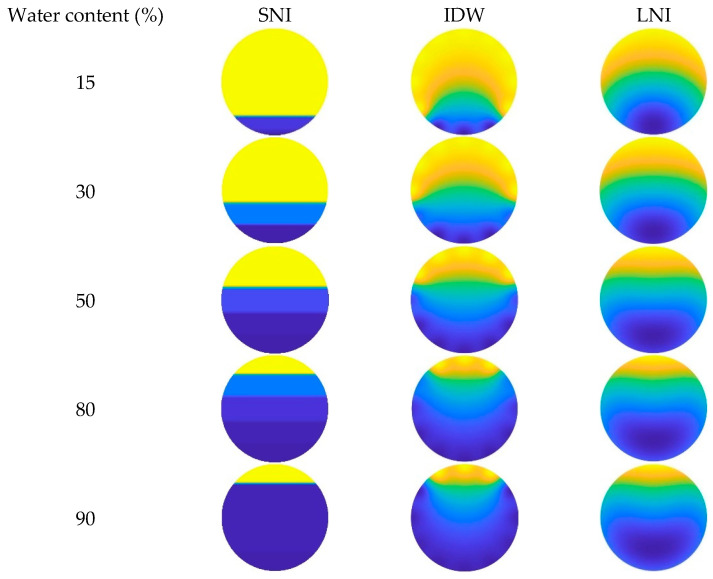
Imaging results for various water contents obtained without CAT at a total flow of 300 m^3^/d.

**Figure 27:**
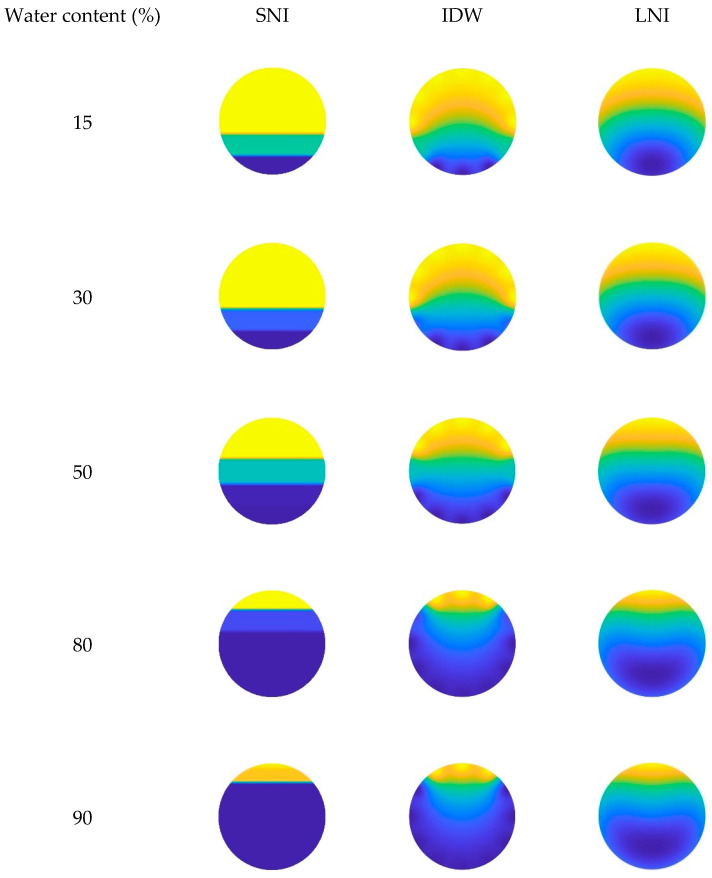
Imaging results for various water contents obtained without CAT at a total flow of 500 m^3^/d.

**Figure 28:**
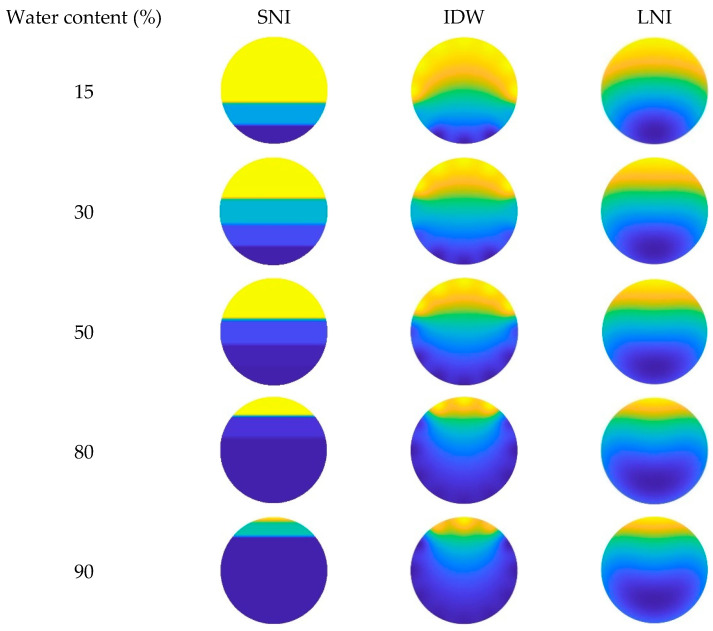
Imaging results for various water contents obtained without CAT at a total flow of 700 m^3^/d.

In this paper, the simple linear interpolation algorithm, the inverse distance interpolation algorithm, and the Lagrange interpolation algorithm are examined, and their imaging performance under different conditions is analyzed. The imaging results of the three interpolation algorithms, taking into account the CAT position in the wellbore, are shown in Figure 23, Figure 24 and Figure 25. Additionally, the imaging effect diagrams of the three interpolation algorithms without considering the CAT position in the wellbore are presented in Figure 26, Figure 27 and Figure 28. Specifically, yellow indicates the gas phase, and blue indicates the water phase. The color gradient between yellow and blue reflects the water holdup level across different regions, where lighter shades indicate lower water content and darker shades represent higher water content. These visualizations illustrate the performance of the different algorithms in measuring fluid water holdup under actual experimental conditions, particularly in the complex environment of multiphase flow. The differences in the algorithms’ ability to capture the characteristics of fluid distribution are clearly observable.

As shown in Figure 23 and Figure 26, when the total flow is 300 m^3^/d, the flow pattern in the simulated wellbore predominantly exhibits stratified flow with varying water content conditions. In this flow pattern, the fluid interface between the gas and water phases is distinct, forming a typical stratified structure. From the imaging results, the simple linear interpolation algorithm performs the best. It accurately captures the characteristics of stratified flow, with the imaging effect closely matching actual experimental conditions, particularly in presenting the gas–water interface across varying water content levels. This indicates that the simple linear interpolation algorithm is highly applicable under low-flow conditions and can reliably reflect the fluid distribution in the wellbore. The imaging performance of the Lagrange interpolation algorithm is generally satisfactory. Although it is not as precise as the simple linear interpolation algorithm in some details, its overall performance is acceptable, providing accurate water holdup calculations in most cases. The inverse distance interpolation algorithm performs better under high water content conditions, adequately reflecting the actual situation. However, its performance under low-water-content conditions is less satisfactory, with the imaging results showing significant deviations from the actual experimental conditions. This discrepancy suggests that the inverse distance interpolation algorithm may have limitations in capturing fluid distribution at low-water-content levels, and it struggles to accurately reflect fine variations in the flow pattern.

As shown in Figure 24 and Figure 27, when the total flow is 500 m^3^/d, the flow pattern in the simulated wellbore primarily exhibits stratified flow under low- and medium-water-content conditions. In this flow pattern, the fluid interface between the gas and water phases is clearly visible, displaying a distinct stratified structure. In this case, the simple linear interpolation algorithm performs the best. It accurately represents the characteristics of stratified flow, and its imaging results are highly consistent with the actual experimental conditions. The simple linear interpolation algorithm is particularly effective at capturing the distribution at the gas–water interface, outperforming the other two interpolation algorithms. However, when the water content exceeds 80%, the flow pattern in the wellbore changes significantly, gradually transitioning into dispersed flow. In the dispersed flow pattern, there is no distinct interface between the gas and water phases, and the fluid distribution becomes more complex. At this point, the Lagrange interpolation algorithm shows clear advantages. It effectively captures the characteristics of dispersed flow and accurately reflects the complex distribution of fluids in the wellbore. In contrast, while the inverse distance interpolation algorithm performs reasonably well, it is slightly less effective than the Lagrange interpolation algorithm in capturing the details of dispersed flow. The simple linear interpolation algorithm performs the worst under high water content conditions, as its limitations in handling dispersed flow prevent it from accurately representing the complex fluid distribution.

As shown in Figure 25 and Figure 28, when the total flow reaches 700 m^3^/d, the flow pattern in the simulated wellbore still exhibits stratified flow under low water content conditions, with a clearly visible fluid interface between the gas and water phases. At this point, an analysis of the imaging results shows that the simple linear interpolation algorithm performs best under these conditions. However, as the water content gradually increases to a medium-high level, the flow pattern in the wellbore shifts to dispersed flow. Under dispersed flow conditions, the distinct interface between the gas and water phases disappears, fluid mixing increases, and the distribution becomes more complex. In this scenario, the Lagrange interpolation algorithm performs the best, with imaging results closely matching the actual experimental conditions. Meanwhile, the inverse distance interpolation algorithm also outperforms the simple linear interpolation algorithm in medium- to high-water-content conditions. Although the inverse distance interpolation algorithm is slightly less effective than the Lagrange interpolation algorithm, it still provides more accurate imaging results under dispersed flow conditions, performing better than the simple linear interpolation algorithm in these circumstances.

## 6. Conclusions

In this study, the response characteristics of the CAT under gas–water two-phase flow conditions were thoroughly analyzed. The experimental results demonstrate that when the well deviation is 90 degrees, the CAT provides more accurate water holdup measurements under total flows of 300 m^3^/d and 500 m^3^/d, showing greater consistency with actual experimental conditions. Building on this, the study employed the simple linear interpolation algorithm, inverse distance interpolation algorithm, and Lagrange interpolation algorithm to image and analyze the gas–water distribution in the simulated wellbore under varying flow and water content conditions. By comparing the imaging results across different conditions, the following conclusions were drawn:

(1) When the total flow is 300 m^3^/d, the fluid in the simulated wellbore exhibits stratified flow regardless of changes in water content, with a clearly visible interface between the gas and water phases. Among the three interpolation algorithms, the simple linear interpolation (SLI) algorithm produced the best imaging results, demonstrating the highest consistency with the actual experimental observations. It also yielded the lowest relative error in water holdup calculation under this flow condition, indicating that SLI is best suited for low-flow, stratified scenarios due to its ability to accurately capture the gas–water interface.
(2) When the total flow is 500 m^3^/d, the flow pattern remains stratified under low- and medium-water-content conditions, where SLI still performs better than the other two methods in terms of both image fidelity and holdup accuracy. However, as the water content increases, the flow pattern gradually transitions into dispersed flow. Under these conditions, the Lagrange nonlinear interpolation (LNI) algorithm shows superior performance, closely matching the observed distribution of the gas and water phases. In terms of water holdup calculation, LNI achieves higher accuracy under high-water-content conditions, while the inverse distance weighted (IDW) algorithm also performs well, though slightly less accurately than LNI. Both LNI and IDW offer practical advantages in capturing the gradual and nonlinear transitions of the flow structure.
(3) When the total flow is 700 m^3^/d, the influence of water content on the flow pattern becomes more pronounced. At low water content, the flow remains stratified, and SLI continues to provide satisfactory imaging and holdup estimation. However, with increasing water content, the flow becomes highly dispersed, and both LNI and IDW outperform SLI in reconstructing the complex fluid distribution. Particularly, LNI yields the lowest relative error in water holdup and provides the most accurate visualization of the internal gas–water distribution under these high-flow, high-complexity conditions.
(4) With the continuous advancement of technology, particularly the development of artificial intelligence, neural network interpolation algorithms have gained increasing attention and practical application in solving interpolation problems. With strong generalization and self-learning capabilities, neural networks can automatically extract patterns from complex, nonlinear datasets, making them well-suited for modeling and predicting fluid distributions in wellbore cross-sections. Future research will focus on incorporating neural networks to enhance the fusion and optimization of multiple interpolation results, and on developing intelligent interpolation models with adaptive strategy selection capabilities. Such models will be able to dynamically adjust interpolation strategies based on sensor response characteristics and wellbore flow conditions, thereby improving the accuracy and robustness of imaging results under varying flow regimes.

## Figures and Tables

**Table 1:** Experimental protocol of gas–water two-phase flow.

Well Deviation (°)	Total Flowrate ($m^{3}/d$)	Water Content (%)
30, 90	300, 500, 700	15, 30, 50, 80, 90

**Table 2:** Water holdup response value of each CAT sensor when the total flow is 300 $m^{3}/d$.

Water Content (%)	15	30	50	80	90
1	0.1226	0.1249	0.1311	0.1409	0.1418
2	0.1389	0.1352	0.1405	0.1421	0.1433
3	0.1436	0.1345	0.1455	0.1462	0.1481
4	0.1455	0.1459	0.1465	0.6488	0.7521
5	0.144	0.4445	0.8139	0.9895	0.9956
6	1.0134	1.0155	1.0159	1.0166	1.0171
7	1.0227	1.0235	1.0238	1.0241	1.0245
8	1.0168	1.0142	1.0141	1.0145	1.0144
9	0.1449	0.4563	0.8146	0.9926	0.9963
10	0.1411	0.1452	0.1458	0.6518	0.7439
11	0.135	0.1378	0.1382	0.1383	0.1491
12	0.1233	0.1252	0.1305	0.1315	0.1411

**Table 3:** Water holdup response value of each CAT sensor when the total flow is 500 $m^{3}/d$.

Water Content (%)	15	30	50	80	90
1	0.1309	0.1522	0.1636	0.201	0.2539
2	0.1435	0.1551	0.1672	0.2066	0.2577
3	0.1472	0.1586	0.1691	0.2085	0.662
4	0.1491	0.1599	0.1705	0.8536	0.9515
5	0.1499	0.6353	0.9511	0.996	0.9962
6	1.0145	0.6353	1.0165	1.0171	1.017
7	1.0236	1.016	1.0235	1.024	1.0242
8	1.0143	1.0229	1.016	1.0168	1.0166
9	0.1488	0.6412	0.9489	0.9963	0.9969
10	0.1482	0.1593	0.1715	0.8622	0.9513
11	0.1477	0.1579	0.1699	0.209	0.689
12	0.1338	0.154	0.1651	0.2051	0.2526

**Table 4:** Water holdup response value of each CAT sensor when the total flow is 700 $m^{3}/d$.

Water Content (%)	15	30	50	80	90
1	0.1292	0.1311	0.1325	0.1421	0.1433
2	0.1423	0.1428	0.1439	0.1455	0.1469
3	0.1466	0.1475	0.1491	0.1512	0.1525
4	0.1472	0.1487	0.1495	0.7027	0.8261
5	0.1485	0.5266	0.9127	0.9936	0.9957
6	1.0141	1.0158	1.0162	1.0169	1.0168
7	1.0233	1.0233	1.0235	1.025	1.0253
8	1.0146	1.0151	1.0151	1.0163	1.0166
9	0.1479	0.5327	0.5327	0.9954	0.9955
10	0.1475	0.1489	0.1489	0.7039	0.8329
11	0.1470	0.1424	0.1424	0.1461	0.1477
12	0.1301	0.1316	0.1356	0.1426	0.1439

**Table 5:** Results of different methods for calculating water holdup when the total flow is 500 m^3^⁄d.

Water Content (%)	Integral	SLI	IDW	LNI
15	0.2813	0.3226	0.3327	0.3351
30	0.3689	0.4131	0.4262	0.4159
50	0.5914	0.6624	0.6321	0.6215
80	0.8135	0.8567	0.8469	0.841
90	0.9016	0.9695	0.9482	0.9433

**Table 6:** Results of different methods for calculating water holdup when the total flow is 700 m^3^⁄d.

Water Content (%)	Integral	SLI	IDW	LNI
15	0.2931	0.3469	0.3255	0.3212
30	0.3427	0.3911	0.3806	0.3715
50	0.5576	0.6927	0.631	0.6037
80	0.8243	0.9292	0.8823	0.8696
90	0.9141	0.9895	0.9542	0.9481

## Data Availability

The original contributions presented in this study are included in the article. Further inquiries can be directed to the corresponding author.
